# Nutritional profiling of horse gram through NIRS-based multi-trait prediction modelling

**DOI:** 10.1038/s41598-025-01668-x

**Published:** 2025-05-15

**Authors:** Manju Kumari, Siddhant Ranjan Padhi, Mamta Arya, Rashmi Yadav, M. Latha, Anjula Pandey, Rakesh Singh, Chellapilla Bhardwaj, Atul Kumar, Jai Chand Rana, Kailash Chandra Bhatt, Rakesh Bhardwaj, Amritbir Riar

**Affiliations:** 1 ICAR-Indian Agricultural Research Institute, New Delhi, 110012 India; 2 ICAR-National Bureau of Plant Genetic Resources - RS, Bhowali, Uttarakhand India; 3 ICAR-National Bureau of Plant Genetic Resources, New Delhi, 110012 India; 4 ICAR-National Bureau of Plant Genetic Resources - RS, Thrissur, Kerala India; 5 Alliance of Bioversity International and CIAT, Region – Asia, India Office, New Delhi, India; 6 Department of International Cooperation Research Institute of Organic Agriculture, FiBL, Frick, Switzerland

**Keywords:** Nutrition, Diversity, Chemometrics, MPLS regression, RPD, RSQ, Agricultural genetics, Biochemistry, Genetics

## Abstract

Horse gram (*Macrotyloma uniflorum* (Lam.) Verd.) is an underutilised legume from the Indian subcontinent. Being a nutritious legume, it plays an important role in human nutrition in developing countries like India. Conventional assessment of nutritional traits, are labour and time intensive for screening of huge germplasm, hence alternative and rapid technique for conventional method for the determination of nutritional components of horse gram flour is needed. NIRS can be used for this purpose as it gives rapid and precise results for most of the plant products. In this study, a highly diverse collection of 139 horse gram accessions was utilized to generate reference data. Prediction models were developed for protein, starch, TSS, phenols, and phytic acid using MPLS regression method with spectral preprocessing using SNV-DT to remove scatter effects and baseline noise. Models were optimized for derivatives, gap selection, and smoothening and evaluated using different statistics including RSQ, bias and RPD. The RSQ and RPD for the best fit models obtained were protein (0.701; 1.85), starch (0.987; 4.03), TSS (0.800; 4.06), phenols (0.778; 2.15) and phytic acid (0.730; 1.88) indicating developed models are good for screening large number of germplasm collections and market samples. Statistical analyses, including paired t-tests, correlation, and reliability assessments, validated the strength of these models. This study represents the first report introducing a rapid, multi-trait evaluation approach for horse gram germplasm, highlighting its high predictive accuracy for pre-breeding applications. High throughput germplasm screening can be done through these developed models to identify trait-specific germplasm, which can be recommended to develop healthy products and thus can also be recommended for production in the farmer field simultaneously.

## Introduction

Leguminous crops have been acknowledged for their nutritional value as plants that can complement cereals in human diets, especially in developing nations^1^. Horse gram (*Macrotyloma uniflorum* (Lam.) Verd.) is such an underutilised legume indigenous to Indian subcontinent^2^, belongs to the tribe—Phaseolae, sub-family—Faboideae and family Fabaceae^3^. Horse gram is a minor legume in India, cultivated on 0.32 million hectares of land^4^, contributing 1–2% of total pulse growing area. Currently, it ranks fifth among the most extensively grown grain legumes in India and is considered exceptionally resilient. Additionally, it serves as a vital source of vegetable protein for hundreds of millions of rural residents on the subcontinent^5^ especially in the Southern Peninsular India. The major horse gram producing Indian states are Karnataka, Andhra Pradesh, Tamil Nadu, Odisha, Maharashtra, Chhattisgarh, Bihar, Jharkhand Madhya Pradesh, Uttarakhand and Himachal Pradesh.

Horse gram is now emerging as a potential future food legume, due to its high nutritional quality and adaptability to harsh climatic conditions which could contribute significantly to global food and nutritional security^6^. It contains high protein (18–29 g/100 g), carbohydrates (57.2 g/100 g), total dietary fibre (16.3 g/100 g), minerals (3.2%), and vitamins like thiamine (0.42 mg), riboflavin (0.2 mg), niacin (1.5 mg) and Vitamin C (1 mg/100 g)^4^. Apart from nutritional and health-promoting effects, horse gram also possess some anti-nutritional factors like phytic acid (1.02 g/100 g), polyphenols (1.43 g GAE/100 g), oligosaccharides (2.68 g/100 g) which restricts its large amount utilization as human food^7^. While it plays a significant role in traditional diets as a vital source of protein, fiber, and bioactive compounds, its potential as a future food legume remains underexplored in modern agricultural research. Despite its resilience and nutritional value, horse gram has been overlooked in mainstream crop improvement and biochemical profiling studies. Current research trends in legumes have leveraged advanced tools like near-infrared spectroscopy (NIRS) to accelerate the assessment of nutritional traits^8^.

It is a non-destructive, quick approach that does not require any chemical reagents for the quick estimation of different organic compounds in different food forms like grains, flours, fruits, vegetables and meat through prediction modelling^9^ Samples could be evaluated directly regardless of their physical status, due to the high penetration and scatter efficiency of NIRS^10^. Conventional biochemical methods for assessing nutritional traits, such as Kjeldahl for protein estimation or colorimetric assays for phenols and phytic acid, are widely used but are labor-intensive, time-consuming, and require extensive sample preparation, well trained analyst and costly reagents. These methods also involve chemical extraction, making them destructive and impractical for large-scale germplasm screening and also adds to environmental costs In contrast, NIRS offers a rapid, non-destructive, and cost-effective alternative, enabling simultaneous analysis of multiple traits within seconds. Compared to standard wet chemical analysis per-sample cost of estimation is negligible, elimination of hazardous chemicals, and reduced labour requirements make it a more sustainable choice for high-throughput analysis. In this study, the accuracy of NIRS models was validated through statistical analyses, including paired t-tests (p > 0.05), high RSQ_external_ values, and low SEP(C) values, demonstrating strong predictive performance. Given its efficiency, accuracy, and economic feasibility, NIRS emerges as a robust tool for evaluating horse gram germplasm, facilitating trait-specific selection for breeding programs and value-added product development. Theoretically, choosing samples that cover a data set’s spectrum, variation range should be adequate; however, the calibration should be taken into account for all types of variation, not only for genotypic variation^11^. Reference data, generated through laboratory method is primarily required to establish the calibration and validation model of the samples. It is dependent on the absorption in near infra-red area of the electromagnetic (EM) spectrum of molecular overtone and combination vibration of hydrogen groups X–H (X = C, N, O) (from 750 to 2500 nm)^12^. Spectral pre-processing methods such as derivatization, standard normal variate and detrend (SNV-DT), multiplicative scatter correction (MSC), weighted and inverse MSC, along with other techniques, are commonly employed to mitigate light scattering effects. Multivariate regression methodologies like partial least squares (PLS), principal component regression (PCR), multiple linear regression (MLR), and modified partial least squares (MPLS) are applied to produce reliable and efficient models^11^.

NIRS has been widely applied for high-throughput biochemical profiling in many cereals, legumes, oilseeds, tuber crops, etc. Analysis of multi-nutritional traits in brown rice^12^, multi-nutritional traits in cowpea^13^, protein content in mung bean^14^, nutritional quality of dual-purpose cowpea^15^, glucosinolate content in mustard^16^, multi-nutritional traits in lablab bean^17^, and the multi trait nutritional profiling of pearl millet^18^, are some of the recent applications of NIRS in biochemical profiling of crop plants. However, its application in horse gram remains largely unexplored, with existing studies limited to proximate composition or traditional wet-lab methods, which are time-consuming and less scalable. To the best of our knowledge, no studies have developed NIRS-based predictive models for multiple biochemical traits in horse gram, such as protein, fiber, starch, and phenolic compounds. This gap hinders comprehensive agrobiodiversity assessment and the potential integration of this legume into diverse diets.

Our study addresses this gap by creating predictive models using NIRS for biochemical traits in horse gram. This approach not only enhances our ability to evaluate its nutritional profile but also supports on-field agrobiodiversity assessments, contributing to its valorization in sustainable agricultural practices and nutritional planning.

## Materials and methods

### Sample collection and preparation

One hundred thirty-nine accessions of horse gram belonging to different agro-ecological environments were obtained from National Gene Bank, ICAR-NBPGR, New Delhi, India. Accessions were selected based on the information recorded in passport data and variability observed in grain characters. The selected accessions were grown in the experimental field at ICAR-NBPGR, Regional Station (RS) Bhowali (29.3823° N, 79.5196° E, 1654 m ASL), Uttarakhand, India and ICAR-NBPGR, RS Thrissur (10.5276° N, 76.2144° E, 2.83 m ASL), Kerala, India in augmented block design during kharif-2018 following standard agronomic practices. The standard agronomic practices were followed for sowing time, spacing and irrigation schedules. Fertilizer application rates were standardized based on soil nutrient analysis conducted before the experiment. The pods of physiologically mature plants were harvested, manually threshed, cleaned and dried to moisture content up to 8–10%. The 10 g grains of each genotype from both locations were pooled, cleaned and dried to moisture content up to 8–10%.The dried seed samples underwent grinding, homogenization, and sieving through a 1 mm sieve using a Foss Cyclotec flour mill. The resulting flour was then stored in polypropylene tubes in cool conditions (< 4 °C) for NIR scanning and wet biochemical analysis.

### Reference data for NIRS prediction models

#### Total protein content

The FOSS Kjeltec N_2_ Auto Analyser (FOSS Tecator 2300 Kjeltec Analyser Distiller Unit) was used to assess total nitrogen content using the Kjeldahl technique (AOAC 984.13)^19^. Jone’s factor of 6.25 was used to convert percent N_2_ to g/100 g protein.

#### Total starch content

Megazyme total starch assay kit was used to calculate total starch content according to AOAC 996.11^19^, which uses α-amylase, amyloglucosidase, and glucose oxidase. A UV–VIS spectrophotometer (cat# BT-VS-E) was used to measure absorbance at 510 nm, and the results were expressed in g/100 g.

#### Total soluble sugars

Total soluble sugars were estimated spectrophotometrically using anthrone reagent method^20^. Absorbance was recorded at 630 nm, using UV–VIS spectrophotometer (cat# BT-VS-E) and the results were expressed in g/100 g.

#### Total phenolics

The FCR (Folin–Ciocalteu reagent) assay^21^ was used to calculate total phenols, which includes both oxidation and reduction reactions. A UV–VIS spectrophotometer (cat# BT-VS-E) was used to measure absorbance at 650 nm, and the results were represented in GAE g/100 g.

#### Phytic acid

Megazyme commercial test kit containing phytase and alkaline phosphatase enzymes from Wicklow, Ireland (AOAC 986.11), was used to determine phytic acid content^19^. The results were represented in g/100 g and the absorbance was measured at 655 nm.

### Spectral acquisition

Considering that temperature and moisture levels can influence the absorbance and reflectance of NIR waves, the homogenized samples were left at room temperature (25 °C) for 6 h to acclimate to the surrounding environment. Prior to scanning, calibration of the FOSS NIRS 6500 spectrophotometer was performed with Win ISI Project Manager Software v 1.5, using a mica reference tile (100% white). Approximately 5 g of homogenized samples were placed into a circular ring cup equipped with a quartz window (3.8 cm in diameter and 1 mm thick), scanned, and gently compressed with a rear cover to achieve consistent packing of the samples. The sample was scanned 32 times between 400 and 2500 nm to capture the average spectrum which was used for subsequent analysis to ensure consistency and reduce noise from individual measurements, which was then registered as log (1/R) at 2 nm intervals, where R is the corresponding reflectance. Outliers were detected using Neighbourhood Mahalanobis distance (NH > 0.6) and Global H (GH > 2.5) i.e., spectral distance from the mean spectrum of the population^14^. The proximate of each sample to every other sample in the population was estimated by NH. Abrupt spectra were produced due to scanning error in the sample, which becomes an outlier for any trait. The removal of superfluous spectra from the calibration population was done by GH.

### Calibration and validation of NIRS models

A calibration equation was formulated through multivariate analysis, correlating spectral data with laboratory values using Win ISI Project Manager Software v 1.50. MPLS regression with cross-validation was employed to develop equations using the global equations program on the entire spectrum The spectral data for each nutritional parameter underwent preprocessing using mathematical techniques such as Standard Normal Variate and Detrend (SNV-DT). To create calibration and validation sets, the data from 139 samples were sorted in ascending order to ensure an even distribution of diversity in both sets. Every second value was then selected to form the calibration set. This method is suitable for large datasets with a normal or uniform distribution, ensuring that the calibration sets encompass both maximum and minimum trait values and by doing this a framework has been made for creating calibration models. Hence, a total of 99 samples were utilized for the calibration set, while 40 samples were allocated to the validation set, maintaining a ratio of 2:1 to ensure impartiality in the dataset^12^. Various mathematical treatments, were used however, we got our best prediction equations in the treatments i.e., “2,4,4,1”, “2,8,8,1”, and “3,4,4,1”, to develop models. These treatments were chosen based on their ability to optimize model performance for biochemical traits. The first digit denotes the order of derivative which helps in removing the scattering effect, and highlight subtle feature in the spectral data. . The second digit denotes the gap between data points for derivative, While the third and fourth digit refers to the number of points used for smoothing to minimize high-frequency noise while retaining key spectral features^22^. Different statistical parameters like coefficient of determination (RSQ), standard error of cross validation (SEC(V)), standard deviation (SD) and one minus variance ratio (1-VR) were used to assess the developed calibration equations.

Win ISI Project Manager Software v 1.50 was utilized to compute the SEP (standard error of prediction), which represents the discrepancy between the reference values of the calibration set and the values predicted by the NIRS calibration models. RSQ is employed to illustrate the proportion of variation in reference data accounted for by the variance in predicted data, with higher RSQ values (> 0.750) and lower SEP(C) values indicating the superior performance of models^18^.

### Prediction method assessment of the models

Various statistical metrics were employed to assess the predictive capability of the calibration models, including SEC(V) and 1-VR, RSQ_external_ (coefficient of determination in external validation), bias (difference between predicted and reference values), SEP(C) (corrected standard error of prediction), and RPD (ratio of standard deviation to standard error of prediction) values, along with cross-validation^23^. Following the evaluation of these parameters, the equation demonstrating the best fit was deemed the prediction model.

RPD values served as a measure to assess the accuracy and precision of the developed MPLS models. If the RPD value is less than 1.5, the model is considered unreliable. Conversely, a value between 1.5 and 2.5 indicates the model’s capability to differentiate between high and low values, while a range of 2.0 to 2.5 suggests approximate quantitative prediction. Values falling between 2.5 and 3.0 indicate good quality prediction, and an RPD exceeding 3.0 signifies excellent prediction^23^.

### Statistical analyses

All nutritional compositions were evaluated in triplicates to guarantee precision. Calibration and validation was carried out using Win ISI Project Manager Software v 1.50, which employed diverse mathematical methods derived from spectral and analytical data. Reference and predicted values were compared using the above software with the formulated equation. The model’s accuracy and predictive capability were assessed using global statistical indicators such as RSQ, slope, bias, RPD, and SEP(C). The coefficient of determination (RSQ_internal/external_) was plotted externally to visualize all nutritional parameter graphs. Statistical analysis of the analytical findings was conducted using Jamovi software. To assess the prediction accuracy of the model, a paired t-test was conducted using Jamovi software with a confidence level of 95%. The null hypothesis is rejected if p > 0.05, indicating a difference between predicted and reference values. Conversely, if p < 0.05, the null hypothesis is accepted, signifying no difference between predicted and reference values. Strict parallel analysis was performed to check the reliability of the developed models and the reliability score was calculated by Jamovi software between the predicted and laboratory validated samples.

## Results

### Biochemical data for NIR calibration

**Fig. 1:**
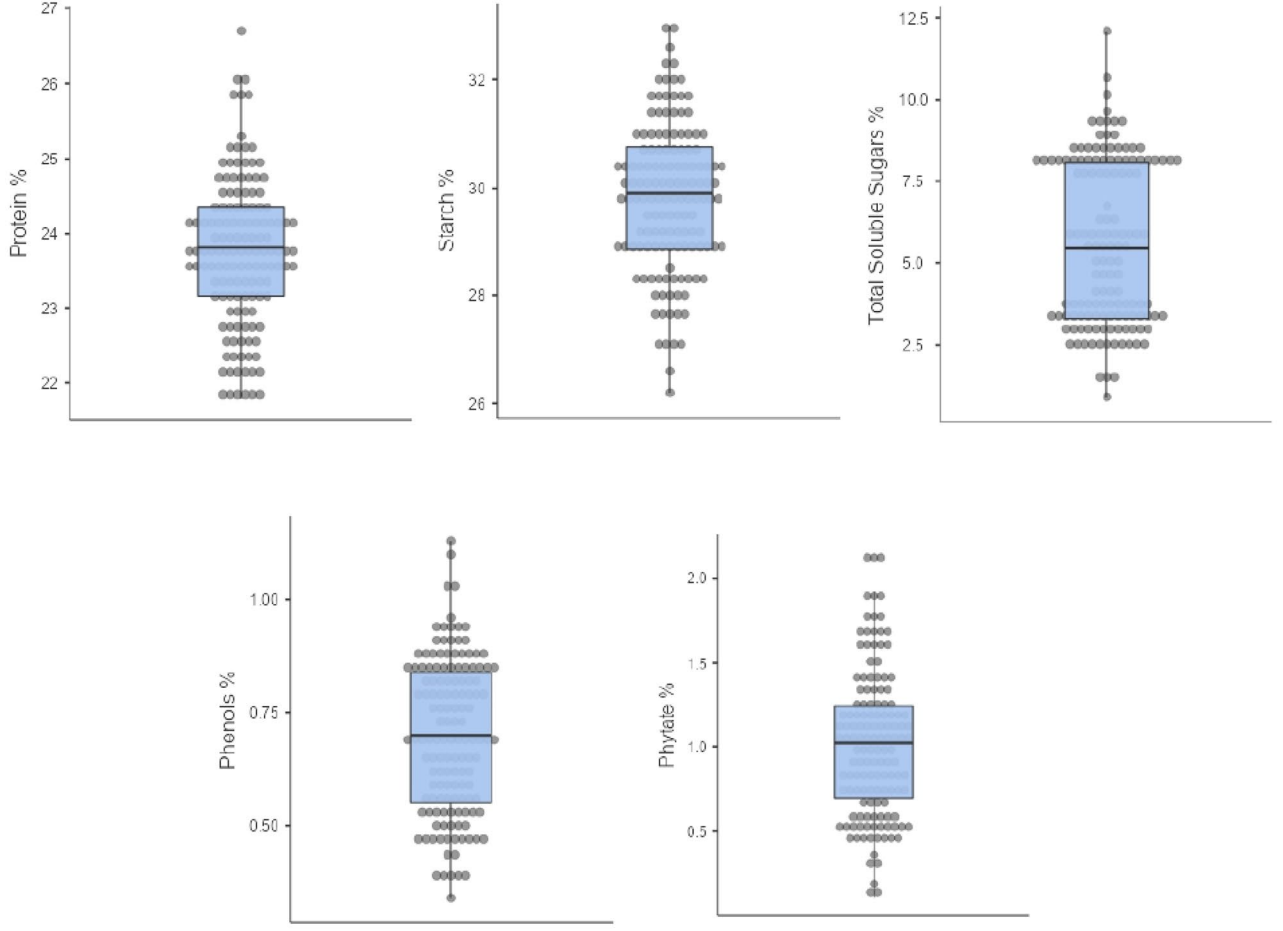
Box and whisker plots of all the biochemical traits of 139 horse gram germplasm.

In this research, we extensively analysed the nutritional content of 139 horse gram accessions by measuring their dry weight. Our statistical examination unveiled significant differences in nutritional composition across these varieties. The descriptive statistics of five biochemical parameters i.e., protein, starch, TSS, phenols and phytic acid of horse gram are summarized in Table 1. The means and standard deviations of protein was 23.7 ± 0.99 g/100 g, starch (29.8 ± 1.36 g/100 g), TSS (5.61 ± 2.5 g/100 g), phenols (0.701 ± 0.17 GAE g/100 g) and phytic acid (1.02 ± 0.44 g/100 g). The variability of data points present in the accessions was visualized as box and whisker plots (Fig. 1).

**Table 1:** Descriptive statistics of total protein, starch, total soluble sugars, phenols and phytic acid.

	Total soluble sugars %	Starch %	Protein %	Phytate %	Phenols %
N	139	139	139	139	139
Mean	5.61	29.8	23.7	1.02	0.701
Standard deviation	2.50	1.36	0.99	0.44	0.17
Minimum	0.860	26.2	21.8	0.110	0.340
Maximum	12.1	33.0	26.7	2.12	1.13

### NIRS spectral assessment

**Fig. 2:**
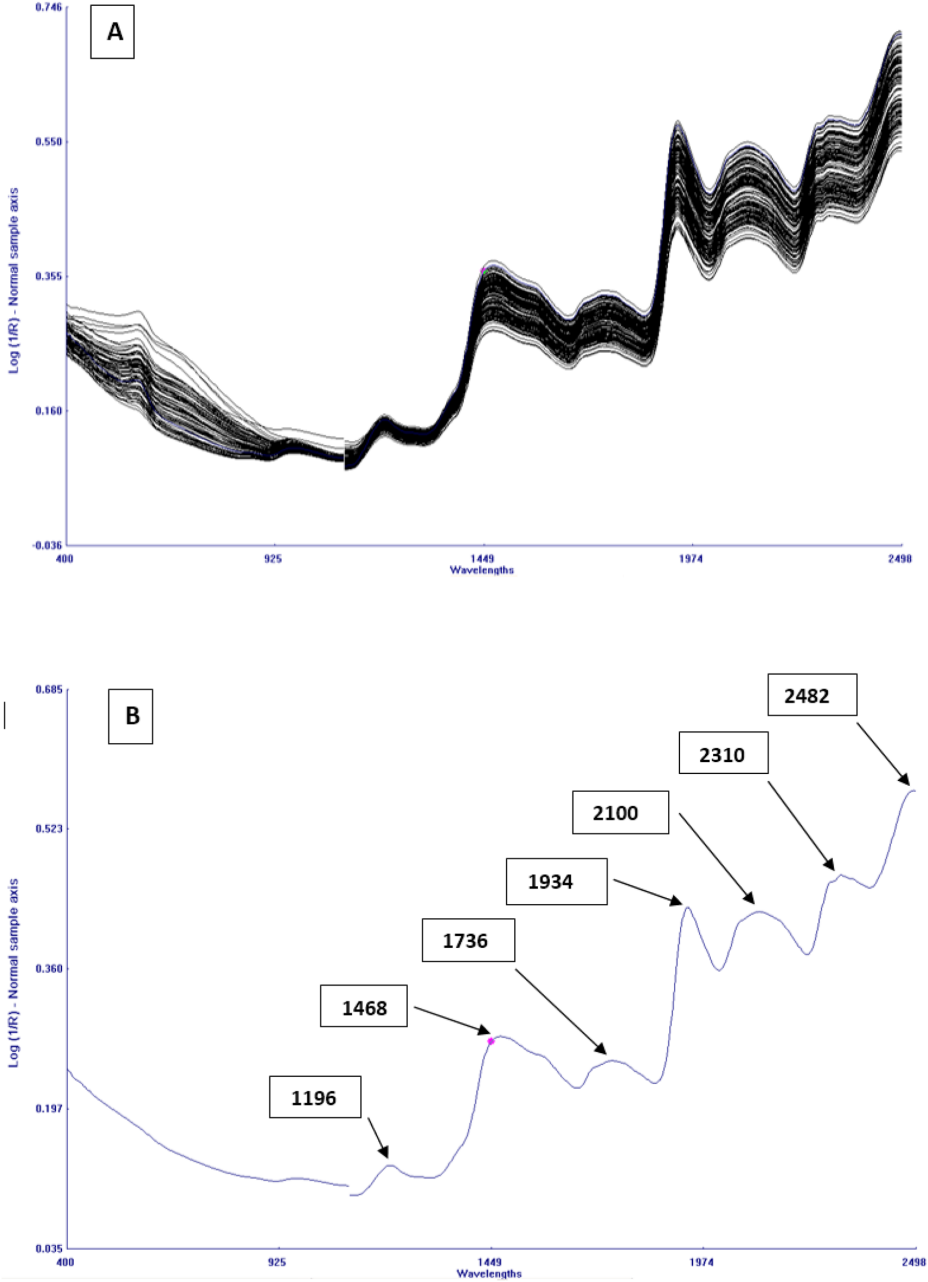
(**A**) Combined reflectance spectra of 139 horse gram germplasm. (**B**) Average reflectance spectrum of horse gram with seven absorption bands.

NIR spectral data of 139 horse gram samples scanned on FOSS NIRS 6500 is shown in Fig. 2A. The spectral profile generated from the instrument is within the range from 400 to 2500 nm, where the main absorption bands were observed at 1196, 1468, 1736, 1934, 2100, 2310 and 2482 mm as shown in Fig. 2B. These absorption bands are the result of overlapping absorption that corresponds to combination and overtones of vibrational frequencies of N–H, C–H, O–H and C–O in chemical components of the samples in NIR^24^. The information about the chemical composition of the component is provided by the position of absorption bands, while the amount of hydrogen containing groups present is determined by the strength of specific band. The C–H second overtone, which corresponds to aliphatic hydrocarbons, is the cause of weak absorption bands detected at 1196 nm^25^. 1430–1470 nm arises due to O–H first overtone stretch corresponding to hydroxyl phenol groups^26^ Near the peak of 1920 nm, O–H bending/stretching of polysaccharides was found, this group can also be found in 1560–1640 nm which can be allocated to O–H group associated with phytic acid^27^. C–O and N–H stretch, which is found in spectral region between 2000 and 2222 nm corresponds to protein content^28^. Third polysaccharide overtone caused by asymmetric C–O–O stretch gives rise to the peak at 2083 nm. Since horse gram flour contains a small quantity of fatty acids, therefore the peaks about 2304–2352 nm, which defines fatty acids and oils, were ambiguous^29^. The sample composition affects the spectrum properties, which serves as a theoretical foundation for the quick estimation of protein, starch, TSS, phenols and phytic acid^30^.

### Calibration of the model

For all the five biochemical parameters two sets were used, one is calibration set constituting 99 samples and another is validation set comprising of 40 samples. Table 2 summarizes the calibration statistics of five biochemical traits i.e., protein, starch, TSS, phenols and phytic acid in horse gram flour obtained from NIRS calibration. Out of the 99 calibration samples of each trait few outliers were observed which may arise due to scanning errors. Such samples arise as a common outlier across all traits hence were removed at the calibration step. Chemometric methods are essential to model the relationship between NIR spectra and measured analytical constituents^31^. Regression algorithms such as partial least square (PLS), modified partial least square (MPLS) and principal component regression (PCR) are commonly used for model development but only MPLS was used in our study for the generation of equations owing to more accuracy and stability as compared to PLS and PCR^32^. Among spectral pre-processing methods, SNV-DT was used in the present study to avoid any noise in NIRS signal baseline. Different mathematical treatments were used for development of calibration equations for various parameters however mathematical treatments “2,4,4,1”, “2,8,8,1” and “3,4,4,1”, were finalised based on performance in external validation. Second derivatives performed best for starch, TSS and phenols while third derivatives with protein and phytate. In general there is no association for derivative order appropriateness with any specific trait, however, second and third derivatives are most commonly used in NIRS modelling while at times first and fourth order derivatives also shows appropriateness^12,13,18^. RSQ_internal_ for different traits obtained for protein (0.650), starch (0.979), TSS (0.845), phenols (0.734) and phytic acid (0.933) are given in Table 2, for given mathematical treatments “3,4,4,1”, “2,8,8,1”, “2,4,4,1”, “2,8,8,1” and “3,4,4,1” respectively.

**Table 2:** Calibration statistics of 99 horse gram accessions.

Traits	N	Outliers	Range (%)	Math treatment	Mean	RSQ	Slope	SD	SEC (V)
Protein	99	4	20.9–26.6	3,4,4,1	23.8	0.650	1.076	0.918	0.622
Starch	99	2	26.1–33.7	2,8,8,1	29.8	0.982	1.008	1.308	0.063
TSS	99	4	0.86–12.1	2,4,4,1	5.73	0.845	1.023	2.483	1.108
Phenols	99	5	0.20–1.18	2,8,8,1	0.692	0.734	1.000	0.163	0.085
Phytic Acid	99	4	0.11–2.12	3,4,4,1	0.985	0.933	1.000	0.390	0.266

*RSQ* coefficient of determination, *SD* standard deviation, *SEC(V)* standard error of cross validation.

### Validation of the model

**Fig. 3:**
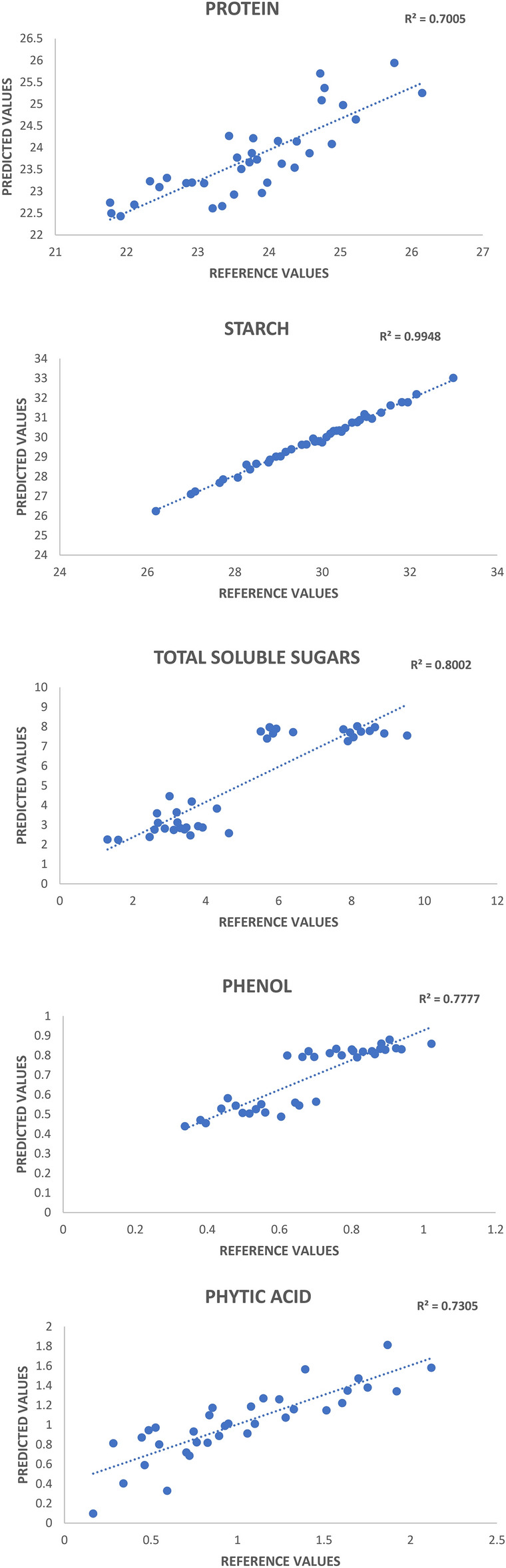
Regression plots of laboratory versus predicted values for all the worked biochemical traits.

The validation phase involved 40 samples to assess the performance of the developed models. Various statistical measures, including RSQ_external_, slope, bias, RPD, and SEP(C), were employed to gauge the accuracy and precision of the models (Table 3). Figure 3 displayed the regression plots of predicted and reference values of the target traits for NIR prediction models. RSQ_external_ for protein was 0.701, starch (0.987), TSS (0.800), phenols (0.778) and phytic acid (0.730) (Fig. 3) indicating a better fit for the respective models. The range for the conventionally calculated values in the calibration and predicted values by the model for all the biochemical traits are summarized in Table 2 and Table 3 respectively. We can note a strong agreement between the calculated and predicted values, indicating a high level of prediction accuracy for the model. The value of slope observed in protein was 0.982, starch (0.982), TSS (1.092), phenols (1.029) and phytic acid (1.215). The slope for protein, starch and phenols is close to one (on rounding off to one decimal point), indicates predicted values for entire range are in proper alignment with laboratory values. The slope for TSS and Phytic acid is close to 1.1 and 1.2 respectively indicating intra and extrapolation with possible slight under estimation at lower boundary and higher estimation at upper boundary. The bias values for phytic acid (0.004), phenols (-0.003) and starch (-0.004) are negligible indicating predicted and laboratory value axis are nearly overlaid above each other, whereas slight negative bias for TSS (-0.059) and protein (-0.028), indicates predicted values axis is slightly shifted on lower side from laboratory value axis, thus possibly slight under estimation of TSS and protein. Ratio Performance Deviation (RPD) value i.e. ratio of standard deviation to standard error of prediction is considered robust test for model usefulness and it ranged from 1.85 to 4.5. Thus, RSQ more than 0.7, slope close to one, bias close to zero, RPD more than 1.8 and p-value more than 0.05 indicates, NIRS predicted values can be reliably used for estimating these traits in horse gram germplasm resources.

**Table 3:** External validation statistics of 40 horse gram accessions.

Traits	N	Range (%)	Math treatment	Mean	RSQ	Slope	Bias	SD	SEP (C)	RPD
Protein	40	21.7–26.1	3,4,4,1	23.7	0.701	0.982	− 0.028	1.101	0.595	1.85
Starch	40	26.2–32.9	2,8,8,1	29.8	0.987	0.982	− 0.004	1.518	0.376	4.03
TSS	40	1.31–9.52	2,4,4,1	5.04	0.800	1.092	− 0.059	2.404	0.592	4.06
Phenols	40	0.33–1.02	2,8,8,1	0.689	0.778	1.029	− 0.003	0.183	0.085	2.15
Phytic Acid	40	0.16–2.12	3,4,4,1	1.02	0.730	1.215	0.004	0.510	0.272	1.88

*RSQ* coefficient of determination, *SD* standard deviation, *SEP* standard error of performance, *RPD* ratio of performance to deviation.

### Statistical analysis between predicted and reference values

A paired sample t-test was conducted to assess the prediction model’s accuracy by comparing the reference values with the predicted values for key traits. This test evaluates whether there is a significant difference between the two sets of values, providing insight into the model’s reliability in predicting the selected parameters. Table 4 presents the paired sample t-test results for protein, starch, TSS, phenols and phytic acid at a 95% confidence interval. The mean differences between the reference and predicted values are minimal, with all p-values exceeding 0.05, suggesting no statistically significant differences. Additionally, the low standard deviation (SD) and standard errors of the mean (SEM) further support the accuracy and consistency of the predicted values across different traits.

**Table 4:** Paired sample t-test at 95% confidence interval.

Pairs	Paired differences							
	Mean	SD	SEM	95% Confidence interval of the difference		t-value	DF	p-value
				Lower	Upper			
Protein reference-protein predicted	− 0.0283	0.162	0.102	− 0.235	0.179	− 0.277	34	0.783
Starch reference-starch predicted	− 0.0085	0.04	0.0181	− 0.045	0.0281	− 0.469	39	0.642
Sugar reference-sugar predicted	− 0.0587	− 0.01	0.184	− 0.433	0.315	− 0.319	35	0.752
Phenol reference-phenol predicted	− 0.0034	0.027	0.0146	− 0.033	0.0262	− 0.235	34	0.815
Phytate reference-phytate predicted	0.00394	0.151	0.0466	− 0.0908	0.0987	0.0846	34	0.933

*SD* standard deviation, *SEM* standard error of mean, *DF* degree of freedom.

Along with the paired sample t-test, correlation and reliability analyses were conducted to further evaluate the model’s predictive performance for nutritional parameters in horse gram germplasm. Table 5 highlights high reliability and strong positive correlations between the reference and predicted values across all traits. The correlation coefficients, ranging from 0.837 to 0.997, indicate a strong linear relationship, while the reliability values (unbiased), ranging from 0.907 to 0.999, confirm the consistency and impartiality of the predictions. These findings underscore the model’s robustness in accurately predicting nutritional traits in horse gram germplasm.

**Table 5:** Reliability (unbiased) test and correlation analysis between reference and predicted values of five nutritional traits in horse gram germplasm.

Serial no.	Traits	Reliability	Correlation
1	Protein (Ref vs. Pred)	0.907	0.837
2	Starch (Ref vs. Pred)	0.999	0.997
3	TSS (Ref vs. Pred)	0.996	0.992
4	Phenol (Ref vs. Pred)	0.932	0.925
5	Phytic acid (Ref vs. Pred)	0.983	0.967

## Discussion

Our findings indicate that NIRS can effectively measure the biochemical traits across a wide range of functional groups. This suggests a positive prospect for the development of global legume/horse gram NIRS-based models. Huge variability was found among biochemical traits in our study where the average protein content is higher than chickpea (18.8 g/100 g), kidney bean (19.9 g/100 g), pigeon pea (20.7 g/100 g), but comparable to black gram (22 g/100 g), lentil (22.5 g/100 g) and green gram (23 g/100 g)^34^. Horse gram is primarily cultivated for its high protein content, which serves as an essential source of vegetable protein in many rural areas. A protein content of 18–27% is considered high for legumes, with 20–25% being optimal for human consumption and animal feed. Thus, identification of high protein accessions could accelerate the quality improvement program of legumes, this is especially relevant for breeding programs targeting food security, as protein-rich crops can help mitigate malnutrition in resource-poor areas. In legumes carbohydrates constitute the major portion (50–70% of the dry matter)^35^. The average starch value (29 g/100 g) is lower than that reported by Bravo et al*.*^35^ (36 g/100 g) constituting of two portions i.e., digestible starch (30 g/100 g) and resistant starch (6 g/100 g). The starch content of horse gram is important as not only contributes to energy value but also provide slow digestible cabohydrates. Horse gram with higher starch content may be preferred for food processing, as it could be used in flour production, offering potential commercial applications^35^. Oligosaccharides, disaccharides (sucrose and maltose) and monosaccharides (glucose, galactose, arabinose, fructose and inositol) constitute the soluble sugars in horse gram, where oligosaccharides constitute the major fraction in the pulses. TSS in our study is comparable to the value reported by Bravo et al*.*^35^ (6.38 g/100 g). TSS levels primarily due to oligosaccharides act as prebiotic by acting as a food to beneficial bacteria in gut, but very high levels also cause flatulence. On the contrary very high content of oligosaccharides is preferred as they add to fermentable sugars, useful in products made from fermented paste or dough. TSS is an important trait for breeders interested in improving the tolerance to abiotic stresses and increasing desiccation tolerance of seeds^35^. Legumes contains some biological compounds which can exert both favourable and unfavourable effects on human body. Undesirable ones are called as anti-nutritional factors (phenols and phytic acid in our study). The value of phytic acid in our study is comparable to Sreerama et al*.*^7^ i.e., phytic acid (1 g/100 g) but lower values in phenols comparatively (1.4 GAE g/100 g)^7^. Phenolic compounds in horse gram are known for their antioxidant properties, contributing to the nutritional value of the crop^7^ besides they also impart tolerance to several biotic stresses in vegetative growth stages^36^. Similarly, phytic acid, binds minerals and aids in accumulating inside the seed but also reduces the bioavailability. However partially hydrolysed phytate act as antineoplastic agent with increase bio availability of minerals^37^.

To ensure unbiased sub-set divisions, all parameters were arranged in ascending order according to their analyzed biochemical parameter. The samples were divided into calibration set and validation set (in the ratio of 2:1)^12^. Samples for validation set were selected after every two samples and the rest samples were filed into the calibration set. Many regression algorithms like PCR, PLS and MPLS can be used however in our study MPLS has been proved to be more accurate and reliable^32^. Before the next factor was computed in MPLS, the NIR residuals acquired after each factor and at each wavelength were calculated and standardized (by dividing them by standard deviation of residuals at each wavelength)^38^. Changes in absorption levels generally occur due to variations in light scattering and path length alterations during sample and light interactions. These complexities pose challenges in linear calibration and spectral interpretation of NIR spectra^39^. Standard Normal Variate (SNV), is employed along with detrend (DT) to correct such signal baseline shifts and minimize noise in the NIRS signal. Mathematical treatments were applied after evaluating various statistical parameters and removing outliers to develop accurate equations.

An external set of 40 samples were used for validating the calibration model. Different statistical parameters like RSQ values, slope, bias, standard deviation (SD), SEP(C) values determine the accuracy and precision of the model. The calibration models demonstrated excellent predictive performance for starch (R^2^ = 0.987, RPD = 4.5) and total soluble sugars (R^2^ = 0.800, RPD = 4.0), indicating that the spectral data captured robust signals corresponding to these traits (Fig. 3). However, the models for protein (R^2^ = 0.701, RPD = 1.85), phenols (R^2^ = 0.778, RPD = 2.15), and phytic acid (R^2^ = 0.730, RPD = 1.88) exhibited moderate predictive accuracy, reflecting limitations in capturing spectral features strongly associated with these compounds (Fig. 3). The moderate accuracy for protein prediction may be attributed to overlapping absorption bands in the NIR region particularly due to oxalates (C═O) and polyphenols (C═C, C═O) stretch with amide (O═C═NH_2_ ↔ O═C─NH_2_) which are comparatively very high in horse gram compared to other legumes^40^. Phytic acid’s spectral signal is primarily associated with phosphorus bonds, which are weaker and less distinctive in the NIR region compared to macronutrients like starch or sugars. This can reduce the specificity of NIR predictions for this trait. Phenolic compounds exhibit complex spectral behaviour due to their diverse chemical structures and potential interactions with other components in the matrix. This complexity can challenge the development of robust predictive models. The combined approach of using normalise spectra with non-linear modelling approaches such as ANN, deep learning methods like DNN, CNN, LSTM and may improve the predictive capacity by resolving spectral overlaps in absorption bands for different functional groups^41–43^. Highest RSQ_external_ was found in starch, followed by TSS, phenols, phytic acid and protein. Similar RSQ_external_ value was found in starch (0.997) and phenols (0.706)^13^ in cowpea germplasm. Towett et al*.*^44^ reported a higher RSQ_external_ of 0.93 for crude protein in cowpea leaves may be due to the higher and wide concentration of protein present in the leaves than in seeds, while Pande and Mishra^27^ obtained an RSQ_external_ of 0.97 for phytates in green gram seeds using FT-NIRS. In other crops Lopez-Calabazo et al*.*^45^ reported R^2^ value of 0.904 for sugar content in lentil, Kjusuric et al*.*^46^ reported R^2^ value of 0.840 for phenols in berry fruits which is approximately similar to our findings. The slope represents the change in predicted values corresponding to a unit change in reference values. An optimal slope value would be 1, with values near 1 indicating a precise model. The value of slope observed in protein was 0.982, starch (0.982), TSS (1.092), phenols (1.029) and phytic acid (1.215). Similar slope values were reported by Padhi et al*.*^13^ in protein (0.903), starch (0.997) and phenols (1.179). Similar values of slope were reported in pearlmillet, cowpea rice and chickpea by Tomar et al*.*^18^ John et al*.*^12^ and Bala et al*.*^47^ respectively. Bias serves as a crucial indicator of the resemblance between reference and predicted values, ultimately influencing the model’s accuracy where the ideal value of the bias should be zero^48^. In our study phytic acid exhibited positive bias values denoting the slight overestimation of the parameter while the remaining parameters exhibited negative bias values denoting the underestimation of the parameters. By carefully selecting mathematical treatments tailored to each trait’s spectral characteristics, the models achieved improved predictive accuracy for most traits. However, traits with moderate prediction accuracy (e.g., protein) may require further optimization, such as dynamic adjustments of derivatives, gap and smoothening or use of deep learning models. Apart from RSQ values, RPD determines the prediction accuracy of the models, which is calculated as the ratio of standard deviation with standard error of prediction between predicted and reference values. As given in Table 3 and Fig. 3, starch (4.03) and TSS (4.06) showed RPD values greater than 3, indicating an excellent predictive capacity of the model. Phenols (2.15) demonstrated an RPD value falling between 2 and 2.5, indicating a very good quantitative prediction capability of the model. Protein (1.85) and phytic acid (1.88) have displayed RPD values between 1.8 and 2.0 indicating good prediction ability of the model^39^. As a general thumb rule, the SEP(C) shouldn’t be greater than 1.30 times the value of the SEC (represents the error in the calibration set and is a measure of how well the model fits the calibration data) while creating robust models. The bias should not exceed SEC by more than ± 0.6, the slope should have a minimum value of 0.90, and the minimum RSQ_internal/external_ should be 0.60^18^. These conditions are fulfilled in our study for the development of the models.

NIRS-based prediction models for biochemical traits like protein, starch, and phenols offer transformative potential for horse gram improvement. For breeders, these models enable the selection of trait specific genotypes and breeding lines, facilitating the development of nutritionally superior and agronomically desirable varieties. Non-destructive phenotyping can also support in identification of molecular marker and genomic regions linked to traits through genome wide association studies (GWAS) and quantitative trait loci (QTL) mapping and accelerating breeding for nutritionally superior varieties. For seed producers and food industry, NIRS provides a rapid method for quality control. Availability of good quality seed of improved varieties with better nutrient profile increases farmer income through pricing based on nutrient profile, enables customer in taking informed choices and contributes to nutritional upliftment. Thus, integration of NIRS into the agricultural pipeline enhances efficiency and addresses critical challenges in food security and sustainability.

Further studies on NIRS for horse gram and other legumes offers exciting opportunities to refine and expand its applications. Optimization of mathematical treatments and cumulation of other machine learning techniques like Deep learning, could enhance the predictive accuracy of complex traits like phenols and phytic acid. Broadening NIRS to additional underutilized legumes, present globally may establish a standardized method for assessing their nutritional quality facilitating achievement of food security and reducing global climate change. Expanding NIRS models to predict a wider spectrum of traits, including polyphenols, fatty acids, oligosachharides and micronutrients, could allow for multi-trait profiling, benefiting both breeders and farmers. Lastly, addressing environmental and genotypic variability by developing robust models across diverse conditions will improve their field-level applicability and ensure reliability in real-world scenarios.

## Data Availability

The datasets generated during and/or analysed during the current study are available from the corresponding author on reasonable request.

## Abbreviations

NIRS: Near infrared reflectance spectroscopy
MPLS: Modified partial least square
TSS: Total soluble sugar
RSQ: Coefficient of determination
SEP(C): Corrected standard error of prediction
RPD: Ratio of performance to deviation
